# TRANS-FOODS study protocol: an observer-blinded, randomized controlled study to assess transcutaneous sensitization to peanut protein in adults with and without atopic dermatitis

**DOI:** 10.1093/skinhd/vzag046

**Published:** 2026-06-25

**Authors:** Preeti Khurana, Saerrah Murryam, Harriet Gilman, Katherine Phillips, Faiza Benaouda, Karl P Lawrence, Mazen Aly, Alexandra F Santos, Stuart A Jones, Carsten Flohr

**Affiliations:** Unit for Paediatric and Population-Based Dermatology Research, St John’s Institute of Dermatology, King’s College London and Guy’s & St Thomas’ NHS Foundation Trust, London, UK; Unit for Paediatric and Population-Based Dermatology Research, St John’s Institute of Dermatology, King’s College London and Guy’s & St Thomas’ NHS Foundation Trust, London, UK; Department of Dermatology, Kingston Hospital, Kingston and Richmond NHS Foundation Trust, London, UK; Unit for Paediatric and Population-Based Dermatology Research, St John’s Institute of Dermatology, King’s College London and Guy’s & St Thomas’ NHS Foundation Trust, London, UK; Department of Population Health Sciences, School of Life Course & Population Sciences, King’s College London, London, UK; Institute of Pharmaceutical Science, Faculty of Life Sciences & Medicine, King’s College London, London, UK; Institute of Pharmaceutical Science, Faculty of Life Sciences & Medicine, King’s College London, London, UK; Institute of Pharmaceutical Science, Faculty of Life Sciences & Medicine, King’s College London, London, UK; Department of Women and Children’s Health (Paediatric Allergy), School of Life Course & Population Sciences, Faculty of Life Sciences and Medicine, King’s College London, London, UK; Peter Gorer Department of Immunobiology, School of Immunology and Microbial Sciences, King’s College London, London, UK; Children’s Allergy Service, Evelina London Children’s Hospital, Guy’s and St Thomas’ Hospital, London, UK; Institute of Pharmaceutical Science, Faculty of Life Sciences & Medicine, King’s College London, London, UK; Unit for Paediatric and Population-Based Dermatology Research, St John’s Institute of Dermatology, King’s College London and Guy’s & St Thomas’ NHS Foundation Trust, London, UK

## Abstract

**Background:**

The rising prevalence of allergic conditions like atopic dermatitis (AD) and food allergies is complex. The dual allergen exposure hypothesis suggests that allergen exposure through skin, particularly in those with AD, can lead to sensitization. This may be exacerbated by skin massage which can enhance allergen absorption by opening the cutaneous appendages. The TRANS-FOODS project aims to elucidate the mechanisms underlying transcutaneous peanut allergy sensitization and evaluate novel preventative strategies, including alterations in peanut processing and adapting skincare practices. As part of the project, a randomized controlled study at King’s College London has been set up to investigate whether skin massage facilitates transcutaneous uptake of peanut allergens, particularly in individuals with AD, and whether a barrier-enhancing cream can mitigate this effect.

**Methods:**

We report the study protocol for a single-site, observer blind randomized controlled trial, where healthy adults and adults with AD (*n* = 130) will be recruited and randomized, applying a peanut protein–containing extract to their outer forearm twice daily with/without massage and with/without a barrier-enhancing cream for a total of 4 weeks. The primary outcome is the potential detection of inflammatory cytokines in skin interstitial fluid (ISF), using a novel non-invasive ISF suction device. In addition, we will study whether the intervention enables detection of peanut protein components in retrieved skin ISF and results in increased transepidermal water-loss measurements.

**Discussion:**

To the best of our knowledge, this study will be the first to use a skin massage intervention with a peanut protein–containing solution and a novel ISF extraction device to understand the effects of massage on the immune system and explore the biological signals associated with food sensitization.

**Protocol registration:**

The trials are registered on ISRCTN (ISRCTN12492694) and ClinicalTrials.gov (NCT05407012).

## Teaser

The TRANS-FOODS study aims to investigate mechanisms of transcutaneous peanut allergy sensitization and potential preventative strategies. We report the protocol for a single-site, observer-blinded randomized controlled trial at King’s College London, recruiting 130 healthy adults and adults with atopic dermatitis to apply a peanut protein extract with/without massage and with/without a barrier-enhancing cream for 4 weeks. The primary outcome is detection of inflammatory cytokines in skin interstitial fluid (ISF) using a novel noninvasive suction device. Secondary outcomes include detection of peanut protein in ISF and transepidermal water loss. This is the first study to combine massage with a peanut protein solution and ISF extraction to investigate mechanisms of transcutaneous food sensitization.

Allergic conditions, such as atopic dermatitis (AD) and food allergies, have become increasingly common, particularly in developed nations. In the UK, AD affects around 20% of children, while nut allergies impact over 2% of the paediatric population.^1^

The development of a food allergy is a complex process, with various hypotheses suggesting an interplay between genetics, diet, skin exposure and the gut microbiome.^2^ One of the most researched ideas is the dual allergen exposure hypothesis, which suggests that early exposure to allergens via the skin may lead to sensitization, particularly in individuals with AD, while oral food protein introduction conveys tolerance.^3–5^ A study by Brough *et al*. demonstrated that infants exposed to peanut protein in household dust were at increased risk of developing peanut allergies, especially if they had *FLG* mutations.^6^ There is also growing evidence that skin massage may exacerbate transcutaneous sensitization to allergens.^7^ Animal studies have shown that mice sensitized to peanut via skin exposure experienced anaphylaxis when later exposed through ingestion, but only when the skin barrier was compromised.^8^

Data from the Barrier Enhancement for Eczema Prevention (BEEP) trial^9^ and the Enquiring About Tolerance (EAT) study^10^ suggest that daily moisturizer application may enhance allergen exposure, potentially through contamination with food proteins. Additionally, an earlier UK cohort study of nearly 14,000 children found that applying creams containing peanut oil to the skin of infants with AD significantly increased the risk of developing peanut allergies.^11,12^

One potential approach to mitigate this risk is early-life dietary exposure to food proteins, which may induce tolerance to these proteins. However, as shown in the EAT study, where only 42% of participants were able to adhere to regular feeding of allergenic foods, ensuring consistent ingestion of allergens in infants is challenging.^7^ Therefore, strategies to reduce skin exposure to food proteins are an important additional food allergy prevention measure. However, the design of such interventions is complicated due to our limited understanding of how peanut allergens make contact with the cutaneous immune system, triggering allergic sensitization.

The European Union Horizon 2020-funded TRANS-FOODS project aims to understand the exact mechanisms behind transcutaneous sensitization and to develop and test novel prevention approaches. Here we present the protocol for an intervention study that forms part of the TRANS-FOODS programme of work to examine the immune responses to peanut allergens in individuals with and without AD, with and without skin massage. Specifically, we aim to determine whether peanut allergen components can penetrate the skin through regular massage and whether this effect is amplified in individuals with a compromised skin barrier. We will also investigate whether preapplication of a barrier-enhancing cream can reduce transcutaneous uptake of peanut proteins.

The primary objective of this study is to determine whether inflammatory cytokines, such as interleukin (IL)-4 and IL-13 can be detected in skin interstitial fluid (ISF) after regular massage using a peanut protein-containing extract.

The secondary objectives are:

- To clarify whether this effect is amplified in those with an ­impaired skin barrier (patients with AD vs. healthy control ­p­articipants).
- To test whether peanut protein components present in skin ISF are able to induce activation of basophils in blood of ­donors allergic to peanuts.
- To assess whether the transcutaneous uptake of peanut protein can be reduced by the prior use of a barrier-enhancing cream.

## Patients and methods

This study protocol was designed following the SPIRIT (Standard Protocol Items: Recommendations for Interventional Trials) guidelines.^13^

### Study design

This is a single-site, observer-blinded, randomized controlled study.

### Eligibility criteria

#### Inclusion criteria

Healthy adults and adults with AD (fulfilling the refined Hanifin and Rajka criteria) between the age of 18 and 65 years who can provide written informed consent and have competent use of the English language will be eligible to take part. Individuals must also be willing to comply with all study requirements.

#### Exclusion criteria

Adults with a history of peanut allergy or a positive skin prick test (SPT) to peanut (SPT >0 mm wheal size) at baseline will not be eligible to participate in the study. No regular consumption of peanut products, widespread AD (particularly if this involves the test sites of the forearms) and the inability to provide informed consent will also lead to exclusion.

### Intervention

A total of 60 healthy adult volunteers and 60 adults with AD will be recruited and then randomized to ensure an even split between groups. A peanut protein extract will be used in all participants. The two groups will then be evenly split into the following groups through computer-generated randomization:

Group 1: application of the barrier-enhancing cream 30 min before application of the peanut protein extract ± massage after extract application.

Group 2: no application of the barrier-enhancing cream, just application of the peanut protein extract ± massage after extract application.

**Figure 1:**
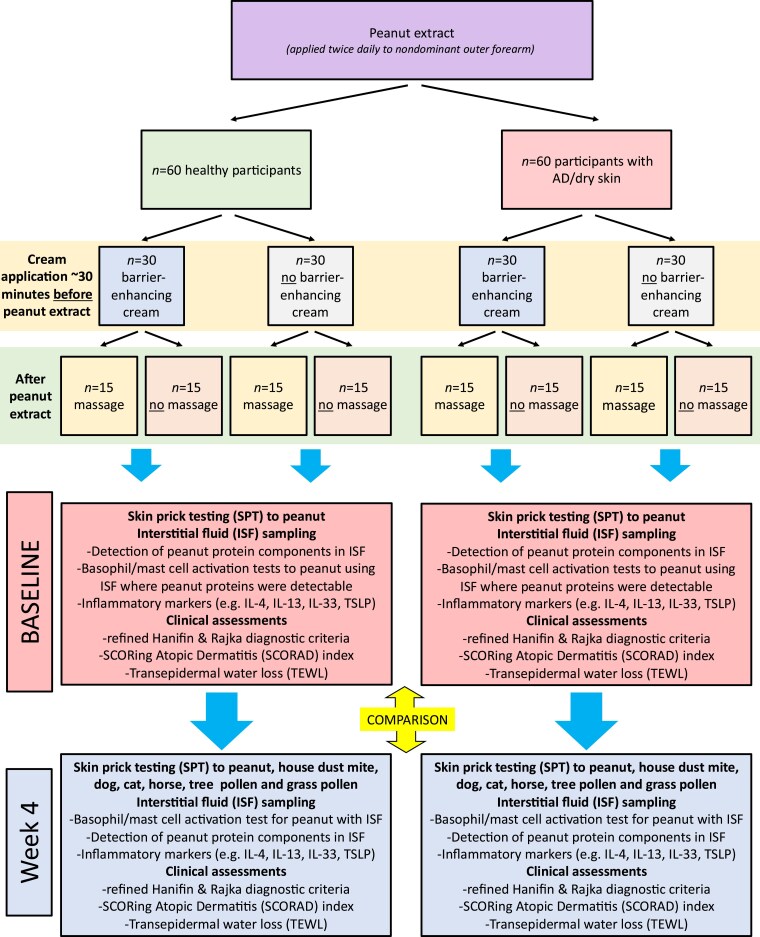
Study flowchart: the peanut extract will be applied to the nondominant outer forearms of each participant, while the peanut extract vehicle will be applied as a control to the dominant forearm. Half of the participants will also have a barrier-enhancing cream applied to the nondominant forearm, 30 min prior to applying the peanut extract. Clinical and sample assessments will be taken at baseline and at week 4. AD, atopic dermatitis; IL, interleukin; TSLP, thymic stromal lymphopoeitin.

This will generate 8 intervention groups, each comprising 15 participants (outlined in Figure 1). Participants will wash their hands and forearms with soap and water before applying the test interventions to the central area of the nondominant outer forearm twice daily with a 5-min massage throughout the 4-week study period. The control formulation (with no protein extract) will be applied without massage to the central, dominant outer arm twice daily as the control. Clear instructions on the massaging technique will be given to all participants, which they will then follow at home using video guidance. Standardized instructions, demonstration videos and written guidance will be used to promote consistent technique and frequency across participants, with weekly text reminders to reinforce adherence and reduce interparticipant variability. Adherence will be monitored using daily record surveys.

Samples of skin ISF will be collected from the control and intervention application sites. Transepidermal water-loss (TEWL) measurements will be taken from the inner forearm. SPT to peanut extract will be performed at baseline to ensure participants are not allergic to peanuts. All baseline measures will be repeated after the 4-week intervention period (Figure 1). Details of the study procedures are outlined below.

### Study procedures and analysis

#### Skin interstitital fluid sampling

Before the study, the potassium phosphate buffer and devices will be autoclaved. Each participant will have their own allocated device. Before use for a different participant, the chambers will be autoclaved. This study will be conducted under standardized conditions. Before commencing the study, participants will be given time to adapt to room conditions and the same investigator will perform all measurements. The skin will be wiped and cleaned using 70% ethanol and tissue. For each participant, the designated skin area will be marked with a washable marker pen. Two tapes will be applied and removed sequentially to remove any skin debris or protein. The skin will again be wiped and cleaned using 70% ethanol and tissue. A 150-µL aliquot of 0.1 M potassium phosphate buffer (sterile, pH 7.4) will be added to the chamber and –9.0 PSI (gauge reading) will be applied for 10 min. After this, pressure will be removed, the buffer will be collected by pipetting up and down and transferred into a 1.5-mL sample-collection tube. During the entire process, the area under the chamber will be carefully monitored for any signs of redness, and the procedure will be stopped if any pain or dark redness is observed. This will be repeated a second time on the arm where the intervention will be applied or has been applied, to ensure two samples of ISF are collected. The same procedure will be done once on the control arm. All samples will be stored on dry ice and then transferred to storage at −80°C until analysis.

#### Transepidermal water loss measurement

TEWL will be measured using a noninvasive commercial measuring device, the Biox Aquaflux AF200 condensing chamber probe (Biox Systems, London, UK). The measurement will be achieved by placing the TEWL probe on the surface of the skin on the inner forearm for 1 min. Measurement will be conducted in triplicate, with the mean taken as the true value.

#### Skin prick testing

Before testing, we will confirm that participants have not taken short-acting antihistamines within the past 48 h or long-acting antihistamines within the past 7 days. All SPTs will be conducted using Allergopharma and Lofarma extracts (available from Diagenics, Milton Keynes, UK), along with positive (histamine 0.1%) and negative control (50% glycerol, 50% buffered saline) solutions. Tests will be performed on the inside of the forearm on eczema-free areas (for participants with AD). A small drop of the control solutions and each allergen solution will be applied to the skin, and the surface will be pricked through each drop using a sterile, single-use lancet (Allegropharma, available from Diagenics). Skin test sites will be measured after 15 min. The wheal and flare will be measured after 15 min by recording their widest diameters and its perpendicular measurement, and the mean measurement will be recorded.

If the negative control produces a wheal ≥3 mm, we will reschedule the test after 7 days. If the positive histamine control produces a wheal ≤3 mm, then the test will be repeated immediately. Should the repeat result remain ≤3 mm, the test will also be rescheduled after 7 days.

#### Cytokine analysis

To measure the cytokines within the ISF samples, a multiplex kit [Meso Scale Diagnostics™ (MSD), Rockville, MD, USA] will be used. The assay will be conducted in 96-well plates. The plates will be washed three times with the supplied wash buffer prior to use. The multianalyte lyophilized calibrators from MSD will be reconstituted in 1000 μL of Diluent 41 (supplied with the kit). Subsequent standards will undergo a fourfold serial dilution. After washing the plates, 50 μL of sample or standard, based on the plate layouts, will be added to the plate. The plates will be sealed and incubated with shaking (700 rpm) at room temperature for 2 h. Following this incubation, the plates will undergo three additional washes with 150 μL of the washing buffer. Subsequently, 25 μL of antibody solution will be added to each well, followed by incubation with shaking (700 rpm) for 2 h at room temperature. Subsequently, the plates will be washed three times with 150 μL of the washing buffer. Finally, 150 μL of the read buffer solution will be added to each well, and the plates will be immediately placed in the MSD Meso Quickplex SQ 120 machine. The detection antibodies in the plate are equipped with labels that emit light when electrically stimulated by carbon electrodes at the bottom of each well. Data analysis will be performed using MSD Mesoscale Discovery Workbench™ 4.0 software, yielding the concentration of each cytokine in pg mL^–1^ for each well.

#### Basophil and mast cell activation test measurements

For the basophil activation test (BAT), whole blood collected from participants allergic to peanuts as part of an ongoing study will be stimulated with the ISF samples, peanut extract or controls in parallel, and basophil activation will be measured as the proportion of CD63-positive basophils by flow cytometry. For the mast cell activation test (MAT), a human mast cell line called ‘Laboratory of Allergic Diseases 2’ (LAD2) cells will be sensitized with plasma from participants allergic to peanuts, mimicking the participants’ own mast cells, and subsequently stimulated with ISF samples, peanut extract or controls, and mast cell activation will be measured by flow cytometry as proportion of CD63-positive LAD2 cells. Biologic activity and allergenicity will be determined by the ability of peanut proteins present in the ISF samples to induce CD63 upregulation compared with standard peanut extract tested in parallel and controls.

### Endpoints

The primary endpoint will be the change from baseline in inflammatory cytokine concentrations (interferon-γ, IL-10, IL-12p70, IL-17, IL-1β, IL-2, IL-4, IL-6 and tumour necrosis factor-α) in skin ISF following regular massage with a peanut protein-containing extract.

The secondary endpoints will be the potential detection of peanut protein components in retrieved skin ISF. If detected, we will also measure whether the retrieved peanut protein can activate mast cells and basophils, comparing the study arms. Raised TEWL will also be a secondary endpoint.

### Sample size

Our previous studies indicate that the coefficient of variation of ISF cytokines from ISF is ∼20%, peanut protein in skin samples is ∼8% and BAT/MAT from ISF ∼30% (unpublished data). In all these cases a group size of 15 will provide over 90% power to detect a doubling of concentration levels. Allowing for approximately 8% dropout, 65 participants will be needed in the healthy skin and AD groups (130 total).

### Participant recruitment, data collection methods and timeline

We anticipate that study recruitment will take a year, utilizing a broad range of methods, including social media posts and collaborations with our national patient partner organizations Allergy UK and the National Eczema Society.

Individuals expressing interest are prompted to contact the study team through email, upon which they are sent the ­participant information sheet. Screening of interested individuals is conducted via a phone call. Once eligibility is confirmed, potential ­participants are booked in to obtain consent, for a SPT and their baseline visit, if eligible to take part. Participants will be required to attend two study visits in total, 4 weeks apart. The duration of the intervention per participant will be 4 weeks.

**Figure 2:**
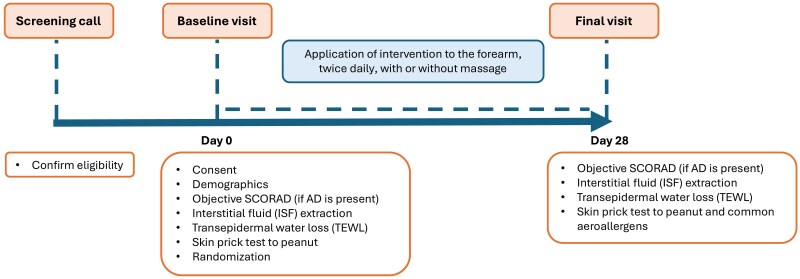
Schematic diagram showing the participant recruitment timeline. AD, atopic dermatitis; SCORAD, SCORing Atopic Dermatitis.

A comprehensive and detailed medical history will be recorded at baseline, including age, ethnicity, any existing skin diseases and concomitant medication. If the individual has AD, disease severity will be determined with the objective SCORing Atopic Dermatitis (SCORAD) index. TEWL readings and SPT results to peanut will be recorded. Skin ISF samples will also be taken, and information on the samples will be recorded. Assessments at baseline will be repeated at week 4 (visit 2; Figure 2).

Participants will be sent a daily record survey electronically through the REDCap database system, enabling the study team to monitor compliance with the study intervention, reinforced through weekly reminder text messages.

To support participant retention all study participants will be offered additional SPT testing to common aeroallergens, including house dust mite, tree pollen, grass pollen, horse, cat and dog. This optional testing will be available to participants only at week 4, during visit 2.

### Allocation

To ensure an even split between the 8 intervention groups, a total of 65 healthy adult volunteers and 65 adults with AD will be randomized 1:1, using block randomization through a code generated by an independent statistician. This computer-generated randomization sequence will be built into the custom-made REDCap database prior to study recruitment. Randomization of participants will be done at the end of the baseline visit by the clinical study team who will have access to the REDCap database.

### Masking

The clinical study team conducting study visits and assessments will not be blinded and will be aware of the participants’ assigned intervention groups. However, laboratory staff responsible for analysing the collected samples will remain blinded to group allocation throughout. Samples provided to the laboratory staff will be anonymized and labelled only with the unique participant identification (ID), date of collection, sample type and study timepoint.

### Data management

The clinical study team will be responsible for data collection under the supervision of the Chief Investigator. Study data will be collected on paper-based case report forms (CRFs) and will be entered into the REDCap database. The daily record surveys completed by participants will be entered directly into the database by them.

All study participants will be allocated a unique study identification number. Participant data collected on paper-based CRFs will be securely stored in a locked room. Data entered into the REDCap database will be securely stored on the King’s College London server. The database will be password protected, and access will be restricted to named study individuals only.

Copies of protocols, CRFs, physiological test results, correspondence, informed consents and other documents relevant to the study will be kept on file by the Chief Investigator’s team at King’s College London and retained for at least 15 years.

### Statistical methods

Comparison of baseline demographic characteristics of study participants across all eight intervention groups will be done using descriptive methods. Adherence to the intervention, including the reasoning behind noncompliance, withdrawal from the study and loss to follow-up, will be summarized. Any adverse events reported by study participants through the daily record surveys or within the visits will also be summarized.

Descriptive and inferential methods will be used for the main analysis. The primary analysis will comprise analysis of covariance to estimate the difference in concentration of inflammatory cytokines in skin ISF between massaging and no massaging patients as per the primary objective by intention-to-treat at week 4. The terms in the model will include the effects of having impaired skin, use of barrier-enhancing cream and massage. Covariates such as gender, ethnicity, baseline TEWL and baseline values of the outcome will be included, with TEWL considered a key mediating factor. Secondary outcomes, including the effect of impaired skin, use of barrier-enhancing cream, and basophil and mast cell activation, and peanut-specific IgE levels, will be analysed similarly. Interaction effects, including those involving massage frequency (categorical and continuous), will also be tested, with significance levels set at 5% for main effects and 10% for interactions. Analyses will account for non-normal data using transformations or nonparametric tests like the Wilcoxon–Mann–Whitney test.

The proportion of participants lost to follow-up after randomization will be summarized in each intervention group. This ­summary will include reasons for loss to follow-up including ­participant withdrawal, withdrawal by the clinical study team and other specified reasons. Baseline characteristics of those missing a week 4 assessment for healthy vs. skin impaired volunteers, cream application vs. no barrier-enhancing cream and massaging vs. no massaging will be compared descriptively to those with complete follow-up at week 4. There will be no imputation for missing data.

### Monitoring

A Study Steering Committee (SSC) has been established for the TRANS-FOODS project and is chaired by the Chief Investigator, providing full oversight of the project, including this trial. The SCC includes members of the clinical study team, all principal ­investigators and their respective teams within the TRANS-FOODS consortium, as well as patient and public involvement representatives. Meetings are every 4 months throughout the project and allow all partners to provide feedback on study findings and plans.

An Independent Data Monitoring Committee (IDMC) has also been set up for the study and is comprised of an independent chairperson, plus two independent members: one who is an expert in the field of paediatric dermatology and one who is an expert in medical statistics. Meetings are held biannually throughout the project, with the Chief Investigator, members of the clinical study team and the trial statistician in attendance. Prior to the IDMC meeting, the study statistician will formulate open and closed reports. The IDMC is responsible for reviewing these and assessing recruitment, interim monitoring of safety and effectiveness, trial conduct and external data. The IDMC will provide a recommendation to the SCC concerning the continuation of the study.

### Adverse events reporting and harms

The study is low risk, and no major adverse events are expected. Nonetheless, the safety of the peanut protein-containing solution and barrier-enhancing cream will be monitored throughout the study. We will ask participants to report any adverse events to us through the daily record survey which they will be sent electronically through the REDCap database system. Participants will also be informed to report adverse events by contacting us directly via the study email or let us know during the study visits. Any adverse events will be recorded on the REDCap database system using the standardized MedDRA protocol.

### Trial registration

The trials are registered on ISRCTN (ISRCTN12492694) and ClinicalTrials.gov (NCT05407012).

### Ethics and dissemination

#### Public and patient involvement

This project was developed through a collaborative effort involving members of the consortium, as well as contributions from allergy sufferers, their families, patient organizations, and charities such as the National Eczema Society and Allergy UK. Patient representatives from the National Eczema Society and Allergy UK are also invited and involved in the SCC meetings as outlined above.

#### Protocol amendments

The original version of this protocol was prepared in January 2023 and there has been one amendment since then. The amendment provided updates on study procedures. As of 28 August 2024, ­v­ersion 2.0 is effective.

If further amendments to this protocol are required, approval will be sought from the King’s College London Research Ethics Committee. The revised version of the protocol will be filed in the Investigator Site File, and corresponding updates will be made to the consent forms and clinical trial registry entry.

#### Consent

Written informed consent will be obtained from all those participating in the study. Prior to this, the participant information sheet and a discussion of objectives, risks and inconveniences of the study will be provided to participants by delegated, trained staff members with experience in obtaining informed consent.

#### Confidentiality

All clinical study staff will ensure to protect the participants’ privacy and consent rights in accordance with the Data Protection Act 2018. Access to study information will be limited to clinical study staff and investigators.

#### Ancillary and post-trial care

It is not anticipated that participants will require post-trial care as they will stop using the intervention after their final visit. However, participants will be advised to contact the study research team via the designated study email address if they experience any symptoms related to the application of the intervention after completing the trial. In such cases, a dermatologist within our team will assess the participant.

#### Data dissemination

The results of the study will be reported and disseminated in peer-reviewed scientific journals and/or conference presentations. Participants will be sent a lay summary of the study results.

## Data Availability

No new data have been generated.
